# A New Clinical Scoring System for Adenoid Hypertrophy in Children

**Published:** 2015-01

**Authors:** Shervin Sharifkashani, Payman Dabirmoghaddam, Maryam Kheirkhah, Rima Hosseinzadehnik

**Affiliations:** 1 *Department of Radiology, Amiralam Hospital, Tehran University of Medical Sciences, Tehran, Iran.*; 2 *Otolaryngology Research Center, Amiralam Hospital, Tehran University of Medical Sciences, Tehran, Iran.*; 3 *Postgraduate, Tehran University of Medical Sciences.*; 4 *Azad University of Dentistry. Tehran, Iran.*

**Keywords:** Adenoids, Endoscopy, Radiography, Signs and symptoms, Sleep apnea syndromes, Snoring

## Abstract

**Introduction:**

Chronic nasal obstruction due to adenoid hypertrophy is a very common disorder. Although the clinical assessment of adenoid hypertrophy is essential, its real value in young children is difficult to evaluate. The purpose of this prospective study was to validate a simple clinical score to predict the severity of adenoid obstruction and to evaluate the relationship between this method of clinical scoring with radiography and nasopharyngeal endoscopy.

**Materials and Methods:**

Ninety symptomatic children were enrolled into this study. The clinical score included difficulty of breathing during sleep, apnea, and snoring. We investigated the relationship between clinical scoring, nasal endoscopy, and radiographic findings.

**Results:**

The clinical score correlated very well with endoscopic findings (P<0.000), but the correlation between the clinical score and radiologic findings (P>0.05) and endoscopic findings and imaging (P>0.05) was weak.

**Conclusion:**

Clinical findings could be used to select children for adenoidectomy, especially when endoscopic examination is not available or cannot be performed.

## Introduction

Chronic nasal obstruction due to adenoid hypertrophy is among the most common health problems affecting children, and adenoidectomy is one of the most common surgical procedures performed in this age group (1). 

Concerns have been raised in the literature regarding the best way to diagnose adenoid hypertrophy in children (2). The real value of a clinical assessment of adenoid hypertrophy is difficult to evaluate in young children. The inaccuracy of patient history reported by some parents and difficulties in approaching young children are examples of subjective drawbacks in the process of clinical decision making (3). Although different objective modalities have been proposed for the diagnosis of adenoid hypertrophy (including mirror examination, palpation, lateral neck radiography, or nasal endoscopy), the role of each of these diagnostic methods is still controversial, and currently there is no comprehensive guideline for assessing adenoidal enlargement (4). 

Because of difficulties associated with the use of the objective methods (such as nasopharyngeal endoscopy) in young children, the development of a reliable scale based on the child’s symptoms to properly evaluate the need for surgical intervention would be of great value for clinicians. 

The purpose of this prospective study was to validate a simple clinical score that was originally used to diagnose sleep apnea in children to predict the severity of adenoid obstruction and to evaluate the relationship between this clinical scoring system and neck radiography and nasopharyngeal endoscopy.

## Materials and Methods

A prospective study was conducted in Amiralam Hospital, Tehran University of Medical Sciences between 2009 and 2012. The study population was pediatric patients with nasal obstruction symptoms for at least 3 months prior to presentation. Patients with obstruction due to septal deviation, concomitant rhinosinusitis, uncontrolled allergic rhinitis, or palatine tonsils greater than grade 2 were excluded. The study protocol was approved by the Internal Review Board of the Institute. Informed consent was obtained from the parents, and parents were present at the time of examination of their children. 

The scoring system used in this study was developed originally for diagnosis of obstructive sleep apnea in children (5). The suggested clinical scoring included the following symptoms: difficulty of breathing during sleep (D), apnea (A), and snoring (S). D and S received a score of 0 (never), 1 (occasionally), 2 (frequently), or 3 (always). Values assigned to A were 0 (if absent) or 1 (if present). 

The final score was derived from the following three-variable function:

Patient score=1.42D+1.41A+0.71S− 3.83

Patients were categorized according to their score into mild (score below −1), moderate (score between −1 to 3.5), or severe (score above 3.5) groups. 

**Fig 1:**
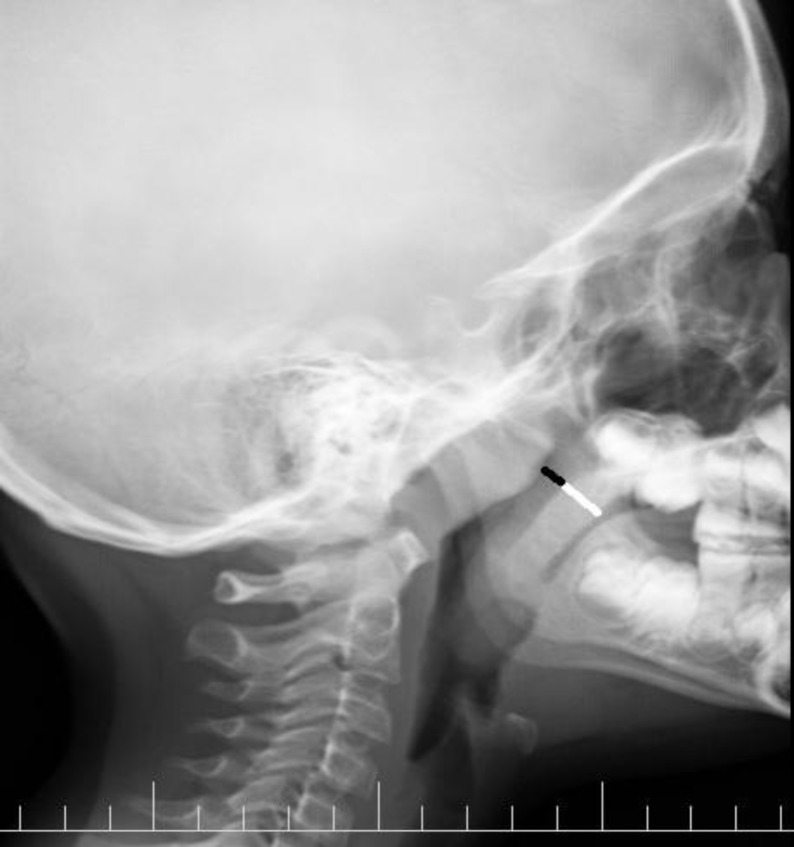
Airway (black line) to soft palate (white line) ratio method of assessing adenoid enlargement on lateral neck radiography as described by Cohen and Konak

A lateral nasopharyngeal X-ray was taken in all patients in an erect position, with the mouth closed and slightly extended neck. The X-ray field was collimated to the nasopharynx with a focus distance of 100 cm, using average exposure factors of 65 kV and 200 mA for 40 to 50 ms. X-rays were acquired on the same day as the nasal endoscopy and under the same setting for all patients. The films were then assessed by a radiologist who was blinded to the physical and endoscopic findings, according to the Cohen and Konak method (6). The airway–to-soft-palate ratio is a comparison between the width of the airway immediately behind the soft palate and the width of the soft palate 1 cm below the hard palate (Fig.1). 

Patients then were categorized into one of three groups: A) Normal (airway-to-soft-palate ratio ≥1); B) Mild-to-moderate hypertrophy (airway-to-soft-palate ratio between 0.5 and 1); and C) Severe hyper- trophy (airway-to-soft-palate ratio <0.5). 

All patients underwent rigid endoscopic nasopharyngoscopy (using 2.7-mm, 0-degree telescope, Karl Storz; Germany) in the clinic. Topical anesthesia in the form of a mixture of lidocaine/phenylephrine was applied prior to endoscopic examination. Patients were asked to maintain a supine position without restraint during the examination; but in some patients it was necessary to immobilize the head. In two patients it was impossible to carry out the endoscopic examination under local anesthesia, so it was performed prior to surgery. Endoscopic examination was recorded and an image with a full view of the choana was obtained. Using this image, an area calculation was performed in an electronic environment using Photoshop CS3 software program (Adobe, USA). The ratio of adenoid tissue to choanal opening was stated as a percentage. Patients were graded based on the degree of obstruction into three groups; in patients in Group 1, <50% of choanal space was occupied by the adenoid tissue, compared with 50–75% in Group 2 and >75% choanal obstruction in Group 3. The physician who performed the endoscopic examination was blinded to the clinical and radiographic findings.

The SPSS software (Version 18.0) was used for the statistical analysis. Spearman’s rank correlation coefficient (a statistical measure of the strength of the relationship between paired data) was used to compare the three methods in pairs. The weighted kappa statistic (a measure for agreement between two scores) was calculated for all possible pairs. To assess the extent of the relationship between two variables, a linear regression analysis (ANOVA) was used. A P-value <0.05 was considered statistically significant.

## Results

**Fig 2:**
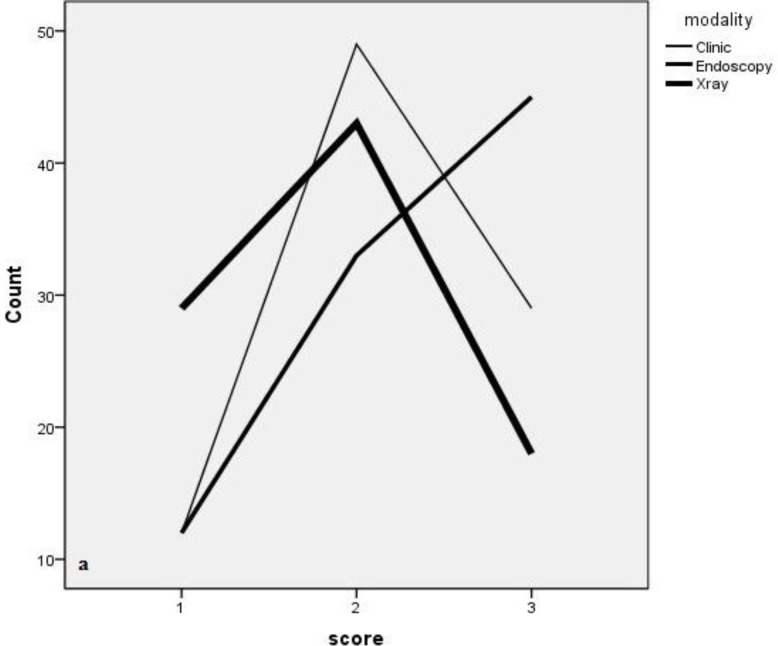
Distribution of categories of degree of severity of adenoid hypertrophy via endoscopic nasopharyngoscopy, lateral neck radiographs, and clinical scoring system

The study group consisted of 90 consecutive children with symptoms of adenoid hypertrophy. The mean age of the children was 141.8 ± 33.45 months, and boys accounted for 56.6% of patients. The mean clinical, endoscopic, and radiological scores are presented in (Table. 1, Fig. 2) compares the distribution of patients according to method of investigation.

**Table 1:** The mean of clinical, endoscopic and radiological scores

**Number of Patients**	90
Mean of Endoscopic Score	2.37± 0.71
Mean of Clinical Score	2.19 ± 0.65
Mean of X-ray Score	1.88 ± 0.71

*Relationship between endoscopic findings and clinical score*

**Fig 3:**
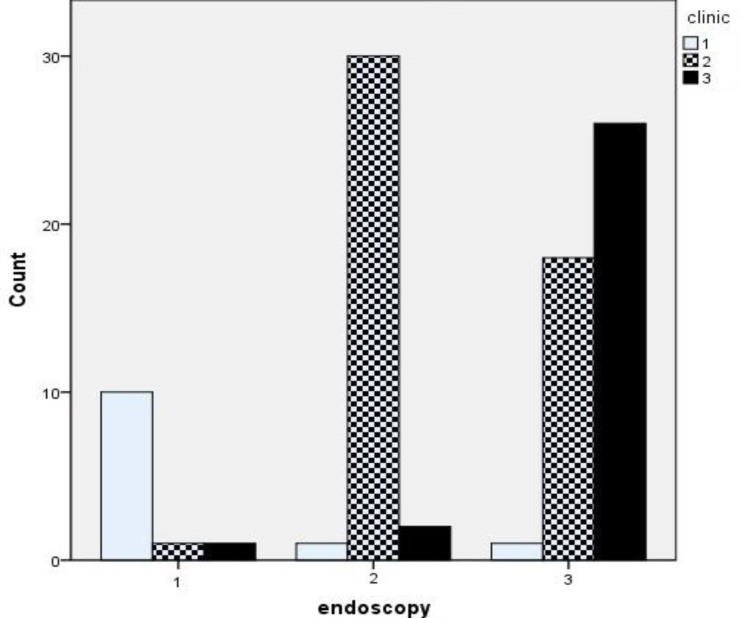
Correlation between clinical score and endoscopic findings

The degree of adenoid hypertrophy as assessed by nasopharyngeal endoscopy and clinical score revealed a significant correlation (Spearman's rank correlation coefficient, r_s_=0.642 [95%, P<0.0001]). The weighted kappa statistics (Kw) comparing the two grading systems was 0.571 which demonstrate moderate agreement (Fig. 3). The linear regression analysis of endoscopic findings against clinical score was significant (P<0.000).

*Relationship between* *clinical score and lateral neck X-ray *

**Fig 4:**
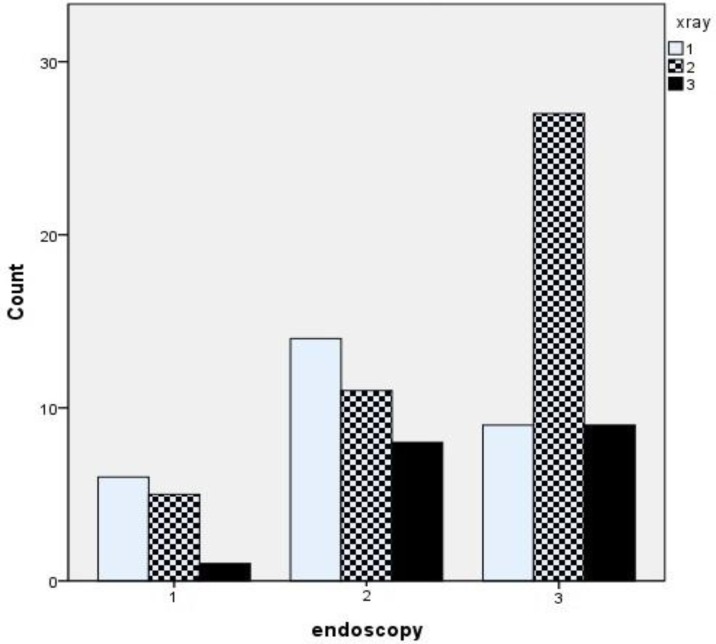
Correlation between endoscopic findings and lateral neck x-ray

Correlation between lateral neck X-ray and clinical score was very weak (Spearman’s r_s_=0.029, P=0.78) and the agreement between these two tools was weak also (Kw=0.054). The regression analysis showed no significant relationship between these variables (P=0.08). As seen in (Fig.4), only 17% of patients with a severe obstruction in clinical score demonstrated severe hypertrophy in the lateral neck radiography.

*Relationship between endoscopic findings and lateral neck X-ray*

**Fig 5:**
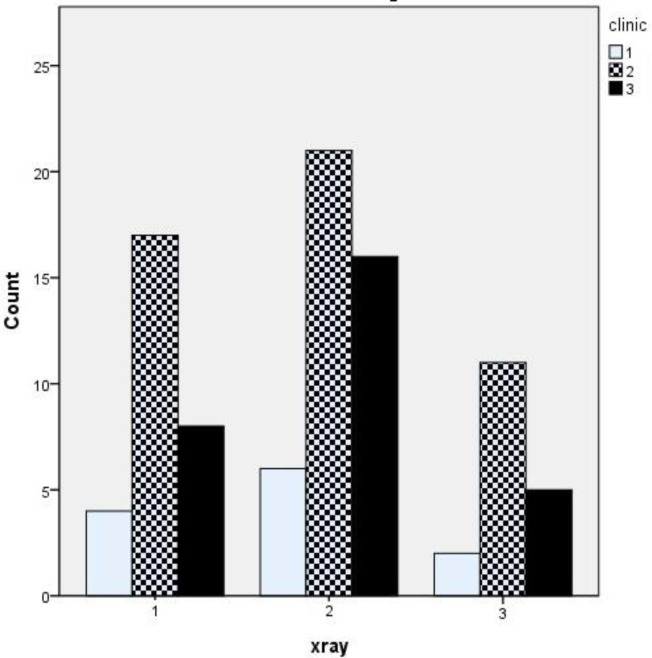
Correlation between radiographical findings and clinical score

Correlation between endoscopic and X-ray staging was weak (Spearman’s r_s_=0.205, P=0.053), with moderate agreement between grading systems (Kw=0.042, P=0.512). The linear regression analysis for these variables was not significant (P=0.6). Eighty percent of patients with severe obstruction on endoscopy revealed normal or mild-to-moderate obstruction upon radiography, while only one patient with normal endoscopy demonstrated severe obstruction upon X-ray (Fig. 5).

## Discussion

Adenoid hypertrophy is a common cause of upper-airway obstruction in pediatric patients and can have a significant influence on the health of the child. Children who have hypertrophic adenoids often exhibit nasal obstruction, snoring, sleep apnea, recurrent otitis media, and craniofacial abnormalities (4). 

Adenoidectomy is one of the most commonly performed major surgical procedures in pediatric patients. Because the adenoid is located in the posterior nasopharyngeal airway, evaluation of its size and the degree of obstruction is challenging. There are several methods to assess the size of the adenoids and their impact on the nasopharyngeal airway. Although each of these approaches have their merits, currently none of them has been accepted by the majority of clinicians as standard practice (4).

Among the diagnostic modalities available, three methods continue to be extensively used in the assessment of adenoid hypertrophy; patient symptoms, lateral airway neck radiography, and endoscopy (7). Objective methods for diagnosing adenoid hypertrophy are valuable in providing information about the need for surgery as well as assessing the outcome of patients after treatment.

Traditionally, soft-tissue lateral neck radiographs have been shown to be effective in the assessment of adenoid size and airway patency (8). Many different radiographic methods have been described in the evaluation of the size of the adenoid tissue, nasopharyngeal soft-tissue size, and airway width on lateral neck radiographs (9). However, there is currently no agreement among authors concerning interpretation of the presence or absence of adenoid hypertrophy in lateral neck X-rays. In a comparative study among four different methods of adenoid measurement on lateral neck X-rays, Wormald et al. concluded that the Cohen and Konak method showed the highest positive predictive value and described this method as a useful diagnostic tool in children with adenoid hypertrophy (10). 

There are some disadvantages associated with lateral neck films, including the exposure of the child to radiation, the lack of standardization in technique and film interpretation, and generation of a two-dimensional image from a three-dimensional structure (2). Furthermore, rotation of the skull and inspiration or phonation during X-ray examination could result in film misinterpretation (11). In our study, we found that correlation of the airway-to-soft-palate ratio with endoscopic examination or clinical symptoms was weak. Although radiographic evaluation of adenoid size has been studied extensively in the literature, the correlation between radiographic findings and clinical symptoms is still very controversial. Some studies show a weak correlation between radiographic findings and adenoid enlargement (1), whereas others exhibit a poor correlation between imaging, adenoid enlargement, and clinical findings (12). 

Nasal endoscopy has been considered as the standard method for the assessment of adenoid size in several studies(10,13,14, 15). It provides a direct anatomical view of the nasopharynx for determining the size of the adenoid and the degree of obstruction of the choanal opening. Nasal endoscopy gives objective and highly accurate results that correlate more closely with the severity of the adenoid hypertrophy than the lateral neck X-ray (1,2,10,11).

Although nasal endoscopy is a reliable and safe diagnostic method, it also has a number of disadvantages. This procedure requires the cooperation of the child, and may be difficult to perform in young children. Furthermore, assessment of the size of the adenoid tissue and choanal obstruction during endoscopy are generally determined based on the subjective analysis of the clinicians, and can reveal discrepancies (9). Calculating the adenoid-to-choanal ratio using digital images and computer programs, according to the method we used, can provide more objective results. Nasal endoscopy is prone to overestimate adenoid size especially for small adenoids (4), and the inter-rater reliability is generally considered poor (8).

The relationship between clinical symptoms and the degree of adenoid hypertrophy has been studied previously (7). Because several of these symptoms (including cough, rhinorrhea, or recurrent upper-airway infections) are non-specific and can occur with other pathologies, other confounding pathologies (such as septal deviation, inferior turbinate hypertrophy, allergic rhinitis, rhinosinusitis, or hypertrophied tonsils) should be excluded before evaluating a child for adenoid hypertrophy. A reliable scoring system to predict the degree of adenoid hypertrophy will be valid only under controlled conditions. Furthermore, therapeutic decision making based on clinical symptoms in pediatric patients has its own difficulties. A major challenge is faced in obtaining a reliable history from the child or credible reports concerning nocturnal symptoms from the parents. 

A wide variety of symptoms have been attributed to the obstructive adenoid. Symptoms such as nasal obstruction, snoring, mouth breathing, sleep apnea, and recurrent otitis media have been the subject of many studies considering their relationship to the severity of adenoidal enlargement (1). The reliability of clinical symptoms in predicting the severity of adenoid hypertrophy is controversial among researchers. In a study by Kubba and Bingham (14), no single symptom in the history of the patient could predict the endoscopic findings. In another study investigating the association of clinical symptoms and the narrowing of nasopharyngeal airway, only snoring showed a significant correlation (7). 

The scoring system used in this study was developed originally to identify children with obstructive sleep apnea, and facilitate selection of children for tonsillectomy and adenoidectomy. This scoring system relies on the most common symptoms encountered in patients with adenoid hypertrophy in the absence of other causing factors (5). These symptoms are nocturnal and related to the direct effect of adenoid hypertrophy on the pattern of breathing and convenience during sleep. As this score is easy to perform and relies on three symptoms only, it would be more acceptable to parents than more complicated scoring systems. 

## Conclusion

Our results show that there is a significant correlation between the nasopharyngeal airway obstruction evaluated by nasal endoscopy and the clinical symptom score, although the correlation between lateral neck radiographic findings and clinical score was weak. The clinical symptom score was found to be a useful and reliable diagnostic tool in patients with suspected adenoid hypertrophy. Based on our findings, this clinical score could be advocated as a parameter for use in selecting children for adenoidectomy, even without adenoid hypertrophy revealed by radiography. This method is particularly suitable for children in whom endoscopic examination cannot be performed or in cases where the facility to conduct endoscopic nasopharyngoscopy is not available.
